# Current Advances in the Development of Hydrogel-Based Wound Dressings for Diabetic Foot Ulcer Treatment

**DOI:** 10.3390/polym14142764

**Published:** 2022-07-06

**Authors:** Viviana R. Güiza-Argüello, Víctor A. Solarte-David, Angie V. Pinzón-Mora, Jhair E. Ávila-Quiroga, Silvia M. Becerra-Bayona

**Affiliations:** 1 Metallurgical Engineering and Materials Science Department, Faculty of Physicochemical Engineering, Universidad Industrial de Santander, Bucaramanga 680002, Colombia; vivragui@uis.edu.co; 2 Program of Biomedical Engineering and Program of Medicine, Faculty of Engineering and Faculty of Health Sciences, Universidad Autónoma de Bucaramanga, Bucaramanga 680003, Colombia; 3 Program of Medicine, Faculty of Health Sciences, Universidad Autónoma de Bucaramanga, Bucaramanga 680003, Colombia; apinzon57@unab.edu.co (A.V.P.-M.); javila28@unab.edu.co (J.E.Á.-Q.)

**Keywords:** hydrogel, diabetic foot, wound dressing, tissue engineering

## Abstract

Diabetic foot ulcers (DFUs) are one of the most prevalent complications associated with diabetes mellitus. DFUs are chronic injuries that often lead to non-traumatic lower extremity amputations, due to persistent infection and other ulcer-related side effects. Moreover, these complications represent a significant economic burden for the healthcare system, as expensive medical interventions are required. In addition to this, the clinical treatments that are currently available have only proven moderately effective, evidencing a great need to develop novel strategies for the improved treatment of DFUs. Hydrogels are three-dimensional systems that can be fabricated from natural and/or synthetic polymers. Due to their unique versatility, tunability, and hydrophilic properties, these materials have been extensively studied for different types of biomedical applications, including drug delivery and tissue engineering applications. Therefore, this review paper addresses the most recent advances in hydrogel wound dressings for effective DFU treatment, providing an overview of current perspectives and challenges in this research field.

## 1. Introduction

Diabetes mellitus (DM) is one of the most common chronic diseases, which is characterized by high blood glucose levels [1]. This occurs when either the body is unable to make insulin, causing hyperglycemia, or when the body cannot use insulin efficiently. In 2019, it was estimated that approximately 463 million people worldwide were affected by this pathology, suggesting that by 2045 this number will increase to 700 million [2]. The multiple complications associated with DM include cardiovascular diseases, blindness, renal failure, and foot ulcers, which lead to morbidity, amputation, and high mortality rates [3]. Additionally, these complications represent a great economic burden for the healthcare system due to the elevated costs of treatment, which in 2017 rounded at about 327 billion dollars. Overall, medical expenses related to the treatment of a diabetic patient have been estimated to average at around 9601 USD per year [4].

Diabetic foot ulcers (DFUs) are a severe condition derived from DM that frequently carries an increased risk of morbidity and early death. It affects around 40 to 60 million diabetic patients worldwide [1], and it is characterized by the formation of chronic wounds, which involve a combination of metabolic disorders, nerve damage, deficient blood flow and biomechanical changes in the lower limbs. DFUs can eventually lead to chronic trauma, infection, and, in the worst-case scenario, lower limb amputation, which is estimated to occur every 30 s globally [2]. Currently, clinical treatments available for DFU management include debridement, pressure relief (“off-loading”), antibiotics, and revascularization [5]. However, in many cases, these therapies fail to ensure full wound repair [6,7], which in turn reinforces the need for novel alternatives that promote tissue regeneration, while minimizing complications in the wound area.

In this context, wound dressing design has progressively gained attention as a potential avenue for the achievement of clinically effective DFU therapies. The ideal dressing for treatment of chronic wounds, such as DFUs, should fulfill several essential requirements [8,9,10]: (1) offer a moist environment that stimulates tissue regeneration; (2) prevent bacterial invasion into the wound; (3) display adequate porosity for gas exchange; (4) promote cell migration, proliferation, and neovascularization; (5) be sterile, biocompatible, and easy to change (or biodegrade). In this regard, hydrogels are polymeric three-dimensional (3D) materials that have been shown promising for successful DFU dressing fabrication [11], since they can provide a moist environment that supports cell proliferation and tissue restoration, promoting scar formation [12,13]. These 3D structures can also exhibit soft tissue-like mechanical properties, as well as be chemically or physically modified to serve as carriers of bioactive agents that can aid in effective wound healing. Therefore, the main purpose of this work was to review current advances in the development of hydrogel-based wound dressing systems, specifically focusing on their fabrication technologies and efficacy in the context of DFU treatment.

## 2. Normal Wound Healing

During normal wound healing, a complex and orchestrated series of events takes place, which results in re-epithelialization and restoration of the injured site. These events must proceed in a regulated fashion through four physiological phases: (i) homeostasis, (ii) inflammation, (iii) proliferation, and (iv) remodeling, as described below:

Homeostasis: upon injury, the immune system swiftly responds via activation of different humoral and cellular cues where antibodies, neutrophils, macrophages, and lymphocytes are recruited to control the infiltration of pathogens that could exacerbate the regular healing process [14]. Additionally, hemorrhaging is mitigated by the formation of a platelet-fibrin clot plug that stops the bleeding and acts as a barrier to avoid interactions between the body system and the outer hostile environment (hemostasis) [15].

Inflammation: the inflammation stage is characterized by swelling and redness around the affected area, due to the removal of damaged cells, growth factors and foreign bodies (e.g., bacteria) from the wound region. Moreover, different inflammatory cytokines, such as interleukin 1 (IL-1), interleukin 6 (IL-6), tumor necrosis factor-alpha (TNF-α), and interferon gamma (IFN-γ) are secreted by neutrophils and macrophages, which further contribute to this inflammatory state as wound repair progresses [16].

Proliferation: when inflammation decreases and wound contraction occurs, revascularization must take place to restore oxygen supply to the wound. At the same time, fibroblasts and epithelial cells migrate to the wound area to induce cell proliferation and differentiation, while simultaneously releasing growth factors, such as epidermal growth factor (EGF), hepatocyte growth factor (HGF), fibroblast growth factor (FGF), and keratinocyte growth factor (KGF), which replace the damaged tissue and the fibrin clot with new extracellular matrix (ECM), promoting re-epithelialization [17,18]. 

Remodeling: after the proliferation phase, collagen fibers reorganize and epithelial cells proliferate and differentiate, restoring the epithelium; at the same time, granulation tissue becomes mature scar tissue, increasing wound resistance, which eventually leads to scar formation and wound closure. Finally, fibroblasts continue to remodel the underlying dermis over a period of several months [19].

## 3. Diabetic Foot Ulcers (DFUs)

**Figure 1:**
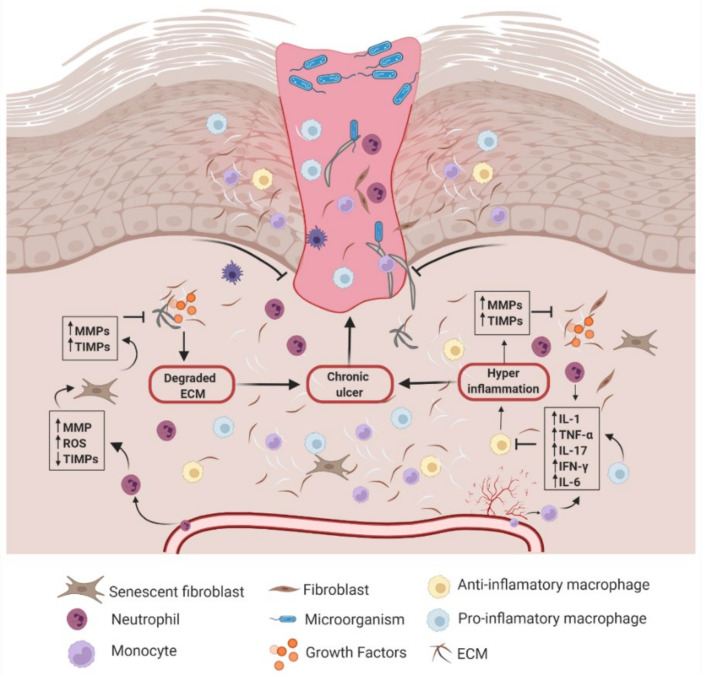
Chronic wound healing process. Diabetic foot ulcers are characterized by continuous inflammation, persistent infections, and necrosis. In these wounds, the balance between Matrix Metalloproteinases (MMPs) and Tissue Inhibitors of Metalloproteinases (TIMPs) is altered, preventing proper remodeling of the extracellular matrix (ECM). Moreover, inflammation is persistent, with high infiltration of immune cells, a response triggered by an increase in interleukins (IL) and pro-inflammatory cytokines associated with cells, such as neutrophils and type I monocytes (pro-inflammatory), which respond to infectious agents. Similarly, there is an increase in reactive oxygen species (ROS), exacerbating the degradation of tissue components. Synergically, these events limit cell migration, angiogenesis, and ECM remodeling, leading to wound chronicity. Created with BioRender.com on 1 June 2022.

In DFUs, the normal healing process is disrupted (Figure 1), producing disorders that demand constant treatment [20]. Thus, the broad range of metabolic dysfunctions in the diabetic patient results in deficient repair of the lesion, which primarily stems from reduced production of pro-angiogenic growth factors and chemokines. Moreover, chronic wounds are commonly characterized by an abnormal and prolonged inflammatory phase, with larger production of metalloproteinases (MMPs) that erode the extracellular matrix at a rate that is too high to allow adequate re-epithelialization and remodeling to occur, even inducing senescence in some cases. Furthermore, MMPs inhibit the action of growth factors, which impairs angiogenesis and limits tissue oxygenation, rendering the wound hypoxic and permanently open. Therefore, open wounds are more prone to pathogenic infections, and infections worsen because of the decreased phagocytosis action induced by hyperglycemia. The combination of all these factors results in even more severe health complications for the patient [18,19,21]. In the face of this challenge, tissue engineering approaches have enabled the design of hydrogel-based systems that function as scaffolds that support proper cell interactions and fill the wound with bioactive agents that promote re-epithelialization, decrease inflammation, accelerate healing, and improve scar formation [22,23].

## 4. Hydrogel Wound Dressings for DFU Treatment

**Figure 2:**
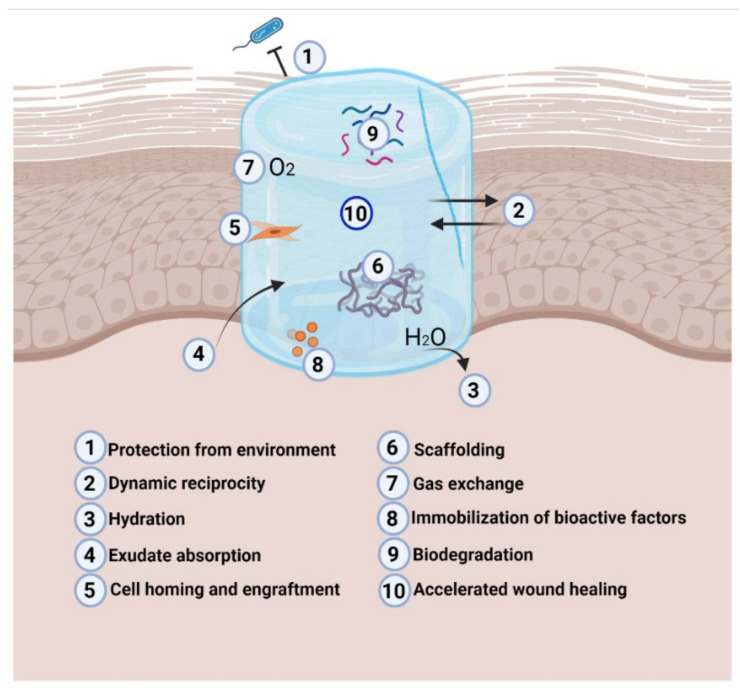
Therapeutic effects of hydrogel dressings during wound healing. Created with BioRender.com on 1 June 2022.

**Figure 3:**
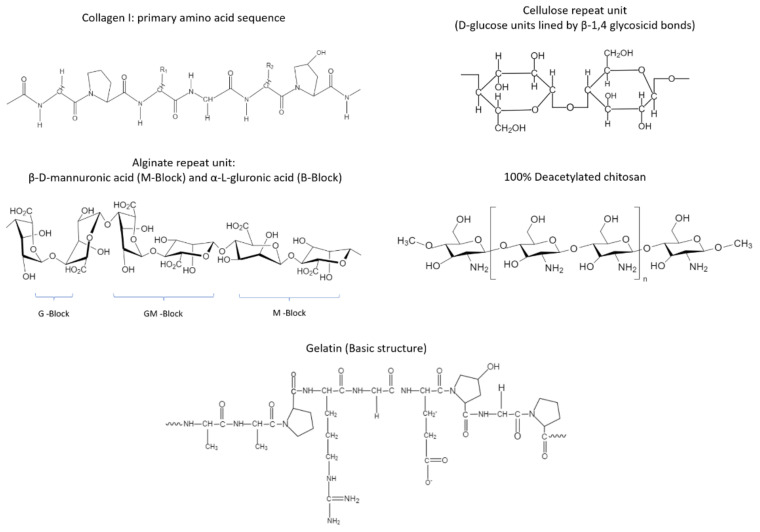
Natural polymers used for the design of hydrogel dressings for DFU treatment.

The unique potential of hydrogels for effective DFU dressing development lies in their remarkable ability to retain oxygen, absorb wound exudate, and maintain the moist environment that supports normal physiological processes in the wound bed [24,25,26]. Moreover, hydrogels have shown promising results as antimicrobial substrates [27] that help avoid tissue death and accelerate regular wound repair, and their porous structure promotes adequate exchange of gases, allowing the wound to breathe throughout wound healing and closure (Figure 2). In terms of their fabrication approaches, hydrogels can be made by the assembly of natural polymers such as collagen [28], gelatin [29], cellulose [30], alginate [31], and chitosan [32] (Figure 3); or they can be produced synthetically by using polymers such as polyethylene glycol (PEG) [33], polyvinyl alcohol (PVA) [34], or polyglycolic acid (PGA). Herein, we present an overview of natural and synthetic polymers that have been recently studied for the development of functional hydrogel systems for DFU treatment and repair.

### 4.1. Natural DFU Hydrogel Dressings 

#### 4.1.1. Collagen and Gelatin Hydrogels

In the context of diabetic wound healing, the therapeutic effect of hydrogels produced from natural polymers such as collagen, alginate or chitosan has been broadly examined in the last few years (Figure 3). Collagen is made of polypeptide chains usually produced by fibroblasts, and it is the most abundant protein found in connective tissue, blood vessels and skin, providing mechanical strength and elasticity [35]. Although there are several types of collagens, collagens type I, II and III are the most predominantly found [36]. Because of its versatile biological activity as well as its biodegradability (thermal or enzymatic) and mechanical properties, collagen has been extensively explored for biomaterial development applications. Specifically, in wound care applications, collagen has been demonstrated to positively impact wound healing by providing structural support and stimulating growth factor release that promotes tissue regeneration. Moreover, its role in MMP deactivation helps maintain favorable biochemical balance and moisture levels in the wound [35].

For instance, Lei et al., evaluated the ability of collagen hydrogels to promote angiogenesis and wound healing in diabetic rat models. Towards this, full-thickness wounds were induced and externally treated with a collagen hydrogel loaded with recombinant human epidermal growth factors. After 14 days of treatment, the rats treated with the fabricated hydrogel showed significantly smaller wound areas, indicating accelerated injury regeneration, relative to the group that did not receive a hydrogel treatment. Regarding angiogenesis, the proposed hydrogel dressing supported endogenous collagen synthesis, as well as the formation of vascularized scar tissue [36]. Moreover, alternative approaches for the development of collagen-based wound dressings have incorporated additional natural or synthetic polymers as well as plant extracts with medicinal properties to enhance the mechanical properties and healing effects of collagen alone in vivo [37,38]. Specifically, the studies performed by Karri et al. reported the fabrication of a hybrid collagen-alginate scaffold that was impregnated with curcumin-loaded chitosan nanoparticles (NPs). Curcumin is a diferuloylmethane found in the Curcuma longa plant, with known antimicrobial, anti-inflammatory, and antioxidant properties [39]. Using a diabetic rat model, the authors evaluated the wound healing effect of the collagen-alginate scaffolds, which were produced by a freeze-drying method. It was observed that the wounds treated with the proposed scaffold displayed improved wound contraction and reduced inflammation, as well as the formation of thick granulation tissue. Positive wound remodeling was further confirmed by proper collagen deposition and epithelial tissue formation [39]. In addition, collagen/chitosan hydrogels have been used as treatment for patients with neuropathic DFU, leading to higher healing rates compared to standard treatment (saline gauze) [38]. Specifically, a Kaplan–Meier survival analysis showed that ulcers treated with the collagen/chitosan hydrogels healed completely after around 12 weeks, whereas ulcers treated with the control took about 21 weeks to heal. In a similar manner, Nilforoushzadeh et al. found that the use of pre-vascularized collagen/fibrin hydrogels on DFU patients resulted in increased hypodermis thickness and an accelerated wound healing process [37].

On the other hand, gelatin is a natural polymer obtained from the thermal hydrolysis of collagen, and it has also been studied for the design of wound dressings (Figure 3). Relative to collagen, gelatin exhibits lower antigenicity [40] and higher degradability, as it can be cleaved by most proteases. Moreover, gelatin is a less expensive alternative to collagen, which preserves the bioactivity required to elicit proper tissue regeneration responses [41,42]. For example, Hsu et al. developed a biodegradable composite hydrogel by combining gelatin and hyaluronic acid (HA) through chemical crosslinking with 1-(3-dimethylaminopropyl)-3-ethylcarbodiimide hydrochloride (EDC chemistry) followed by lyophilization and impregnation with recombinant thrombomodulin (rhTM) rich in epidermal growth factor-like domains. The therapeutic effect of the synthesized hydrogel was examined in full-thickness diabetic wounds on a murine model. After 10 days of treatment, it was observed that a hydrogel formulation containing 0.1% HA and 9 µg rhTM, which also displayed 70% rhTM loading capacity, significantly stimulated collagen production, re-epithelialization, and the formation of granulation tissue, as well as angiogenic response [43].

Further research on gelatin-based dressings, such as the study reported by Zheng et al., explored the use of this biopolymer for the design of injectable scaffolds that could be fabricated in situ to produce dressings that could successfully fill the irregular volume of a lesion through minimally invasive surgical treatments. Specifically, the authors prepared an injectable hydrogel precursor from a gelatin solution loaded with tannic acid, a bactericidal agent, which could then be gelled at body temperature upon blending with gellan through electrostatic complexation [44]. The self-recovery property of this gelatin hydrogel relied on the reversibility of its crosslinks, which rendered a versatile material with in situ moldability to wound beds and self-healing traits. Additionally, the microporous structure of the resulting gel scaffold facilitated cell adhesion and migration, as well as tannic acid delivery into the wound, favoring wound healing and providing a rapid wound closure effect [44]. 

#### 4.1.2. Chitosan Hydrogels

Chitosan is a natural amino polysaccharide obtained from the alkaline N-deacetylation of chitin [45], which has been used in a wide variety of applications as a scaffold in tissue engineering, especially for the fabrication of hydrogel systems due to its low cost and toxicity, ease of production, antimicrobial activity, biocompatibility, and hemostatic potential [46,47] (Figure 3). In addition to this, chitosan is a highly versatile material that can be chemically modified and processed in different forms, such as nanofibers, nanoparticles, nanocomposites, hydrogels, and films, among others. This flexibility favors the development of scaffolds, hydrogels, and bioactive films with tunable properties [48,49]. Overall, chitosan has yielded promising results when implemented for pharmaceutical applications, particularly as drug delivery vehicles with unique potential for wound care applications, as is the case of DFUs [50]. In this sense, chitosan has been recently shown to be an attractive material for the development of hydrogels through additive manufacturing technologies to produce porous 3D scaffolds with specific shapes and bioactivity towards the successful reconstruction of complex tissues. 

For instance, Intini et al., employed a 3D printing approach for the fabrication of chitosan scaffolds with controlled and reproducible structures, as well as mechanical properties that resembled those of native skin [46]. The hydrogel precursor solution consisted of 6% *w*/*v* chitosan in acetic acid, containing 290 mM sucrose to increase hydrophilicity and elasticity of the final scaffold [51]. Upon successful printing, the hydrogel was gelled with 8% *w*/*v* KOH solution and further tested on a rat diabetic wound model. Although no significant differences in wound closure rate were observed relative to a commercial dressing, the proposed hydrogel appeared to provide an enhanced antibacterial effect, a result that was ascribed to chitosan’s intrinsic antimicrobial properties [46]. Moreover, Thangavel and coworkers [52] investigated the influence of natural dressings based on pure chitosan and L-glutamic acid on the wound healing process in diabetic rats. The authors hypothesized that since L-glutamic acid is a known precursor of proline synthesis, its delivery in the wound could stimulate collagen synthesis, and thus, skin regeneration. The proposed hydrogel was prepared through physical crosslinking in 1 M NaOH, in the presence of glycerol as a plasticizer. Comparison of rats treated with gauze dressing, pure chitosan hydrogel, or chitosan hydrogel + 1% L-glutamic acid, indicated the significant therapeutic effect of the latter, as evidenced by complete re-epithelialization after 16 days of treatment (2 cm × 2 cm wounds), in addition to the enhanced levels of collagen deposition and crosslinking that were observed for this group. Furthermore, the positive results from CD31 staining revealed that, indeed, L-glutamic acid promoted new blood vessel formation, whereas a reduction in CD68 levels after 12 days of treatment indicated that the chitosan-L-glutamic acid hydrogel helped regulate the inflammatory response, and therefore, contributed to proper wound healing. Table 1 presents a compilation of recent in vivo studies that have addressed DFU dressing development using natural polymeric sources. 

#### 4.1.3. Alginate Hydrogels

Alginate is a natural block copolymer containing blocks of (1-4)-linked β-D-mannuronic acid (M) and α-L-guluronic acid (G) monomers, typically arranged in three different forms: sequential M residues (MMMMMM), sequential G residues (GGGGGG) and regions of combined M-G units (GMGMGM) (Figure 3). The physical and mechanical properties of alginate can vary depending on how these block sequences are distributed [53].

**Figure 4:**
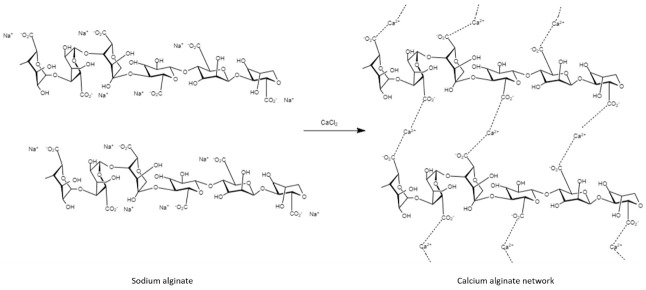
Ionic crosslinking of sodium alginate to produce calcium alginate hydrogels.

Alginate has been widely explored for tissue repair applications involving cell, enzyme, and peptide immobilization, as well as the fabrication of supporting matrices for drug delivery systems, because of its intrinsic ability to allow platelets and erythrocytes to adhere and trigger wound healing responses [54]. Moreover, its hydrophilic nature provides a moist environment that helps reduce scar formation and improves wound re-epithelialization while minimizing bacterial infection. In this regard, Tellechea et al., examined the design of alginate hydrogels (Figure 4) for the encapsulation of human umbilical cord-derived outgrowth endothelial cells (OECs) and the neuropeptides Substance P and Neurotensin, two biological agents with anti-inflammatory attributes. The results from testing in a diabetes-induced mouse model revealed that the alginate hydrogels enabled sustained neuropeptide release during the 10-day period of the study, promoting a remarkable wound size reduction of around 80%. Moreover, the incorporation of vascular endothelial growth factor (VEGF) into the proposed hydrogel further enhanced and accelerated wound healing, as evidenced by a 40% wound size reduction after 4 days of treatment, which suggested a synergistic effect supporting of neovascularization [55]. 

More recently, the use of polydeoxyribonucleotide (PDRN)-based therapies for wound healing has increasingly gained attention, since PDRN has been shown to effectively induce angiogenesis, collagen synthesis, as well as anti-inflammatory responses in the wound bed [56]. In this sense, the studies conducted by Shin et al. demonstrated that PDRN-loaded alginate hydrogels prepared by ionic crosslinking yielded full re-epithelialization of a murine diabetic wound after 14 days of treatment, a process characterized by reduced inflammation and controlled PDRN release [57]. In a similar fashion, therapeutic treatments with edaravone have been elucidated to accelerate DFU wound healing. Edaravone is an antioxidant agent with free-radical scavenging properties that reduces the amount of reactive oxygen species (ROS) present in the wound bed, while preventing vascular endothelial cell injury and inducing angiogenesis in diabetic patients [58].

**Table 1:** Recent in vivo studies on natural hydrogels for diabetic wound healing.

Ref.	Year	Polymer Source and Material	Additional Functional Component (s)	Synthesis Method	Diabetic Model	Therapeutic Effect
[59]	2022	Sodium alginate (2% *w*/*v*) hydrogel	Deferoxamine (560 μg/mL) and copper nanoparticles (200 μg/mL)	Ionic crosslinking with 0.1M CaCl_2_	STZ-induced male C57BL/6 mice	Enhanced antimicrobial effect as well as angiogenesis by upregulation of HIF-1α and VEGF. Reduced inflammatory response.
[60]	2021	Sodium alginate/pectin (5% *w*/*w*) composite hydrogel	Simvastatin (20 mg/mL)	Combined solvent-casting and ionic crosslinking with 0.5% *w*/*v* CaCl_2_	STZ-induced male Wistar rats	Accelerated wound closure due to the presence of SIM, which promoted re-epithelialization, fibroblast proliferation and collagen production.
[61]	2021	Silk nanofiber (1 wt%) hydrogel	Deferoxamine (60 μM and 120 μM)	Concentration-dilution-thermal incubation method	STZ-induced male Sprague−Dawley rats	Enhanced collagen deposition and wound healing rates: 80% on day 14, and 100% on day 21. Improved angiogenic and inflammatory responses.
[57]	2020	Sodium alginate (2–5% *w*/*v*) hydrogel	Polydeoxyribonucleotide (100 μg/mL)	Ionic crosslinking with CaCO_3_	Male C57BLKS/J-db/db mice	Improved re-epithelialization and granulation tissue formation. Increased collagen production and angiogenesis.
[62]	2019	Sodium alginate (1.5% *w*/*w*) hydrogel	Edaravone-loaded Eudragit nanoparticles	Ionic crosslinking with 0.5% *w*/*w* CaCl_2_	STZ-induced male C57BL/6 mice	Downregulation of reactive oxygen species favored accelerated wound healing.
[43]	2019	Gelatin (4% *w*/*v*)/hyaluronic acid (0.1% *w*/*v*) composite hydrogel	Thrombomodulin (9 and 15 μg)	Chemical crosslinking (0.05% EDC)	STZ-induced male C57BL/6JNarl mice	Enhanced granulation tissue formation, re-epithelialization, collagen deposition, and angiogenesis.
[46]	2018	Chitosan (6% *w*/*v*) hydrogel	D-(+) raffinose pentahydrate (290 mM)	Physical crosslinking in alkaline solution (8% *w*/*v* KOH)	STZ-induced female Wistar rats	Increased bactericidal effect and accelerated wound healing.
[52]	2017	Chitosan (2 wt. %) hydrogel	L-glutamic acid (0.25–1.0%)	Physical crosslinking in alkaline solution (1M NaOH)	STZ-induced male Wistar rats	Enhanced re-epithelialization, collagen deposition, and neovascularization.
[39]	2016	Chitosan/starch hydrogel	Chitosan silver nanoparticles (5 ppm Ag in 6.9 mg/mL chitosan)	Reductive alkylation crosslinking	Alloxan-induced male albino rats	Significantly improved wound healing rate. Increased bactericidal response.
[39]	2016	Collagen/alginate (50/50 *w*/*w*) hydrogel	Curcumin (1 wt.%) -loaded chitosan nanoparticles	Chemical crosslinking (EDC)	STZ-induced male Wistar rats	Reduced inflammation. Enhanced cell adhesion and proliferation. Accelerated wound closure.
[63]	2016	Gelatin/hydroxyphenyl propionic acid hydrogel (5 wt%)	Interleukin-8 (IL-8, 0.5 μg/mL) or macrophage inflammatory protein-3α (MIP-3α, 1 μg/mL)	Horseradish peroxidase (HRP)-catalyzed cross-linking	STZ-induced male ICR mice	Increased cell infiltration, re-epithelialization, neovascularization, and collagen deposition.

Fan et al., presented a pioneer work in which alginate hydrogels were used to entrap Eudragit nanoparticles loaded with edaravone. This composite dressing was used on mouse diabetic wounds, and the results after two weeks of treatment indicated ROS downregulation and wound closure, relative to the controls, i.e., free edaravone application (without dressing) or untreated diabetic wounds [62]. Nonetheless, the authors found that significantly positive outcomes could only be attained at low edaravone concentrations in the hydrogel, because high doses of this drug removed too much ROS, which appeared detrimental for wound healing.

### 4.2. Synthetic and Semi-Synthetic DFU Hydrogel Dressings

**Figure 5:**
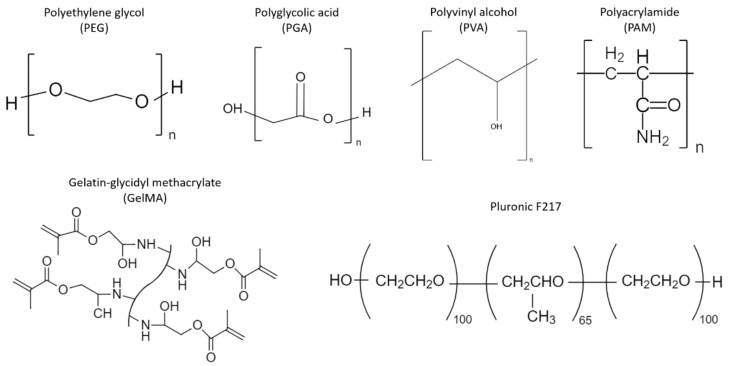
Synthetic polymers used for the design of hydrogel dressings for DFU treatment.

The design of a successful DFU dressing inherently demands the fulfillment of biofunctional as well as structural requirements. Despite the outstanding bioactivity exhibited by natural hydrogels, their mechanical performance and reproducibility still need to be considerably improved. In this context, synthetic polymers are more versatile materials, which display unique properties that can be tailored through controlled physical or chemical processes. As opposed to their natural counterparts, synthetic polymers can be more easily produced at an industrial scale, and their tunability allows for their use in different configurations that favor desired tissue growth [64]. Moreover, because their hydrophilic and hydrophobic domains can be tightly controlled, synthetic polymers can also exhibit more homogeneous structures and higher water absorption capacities. Furthermore, semi-synthetic hydrogels are prepared from natural and synthetic polymers (Figure 5), which combined, result in composite matrices with enhanced biological and material properties. While the natural element provides the desired bioactivity and biocompatibility, the synthetic component facilitates control of hydrogel properties, such as biodegradability as well as mechanical and swelling behavior. This synergistic effect has strengthened the therapeutic potential of hydrogel dressings, and for this reason, semi-synthetic hydrogel systems have been at the core of DFU dressing research for the past few years (Table 2). 

#### 4.2.1. Polyethylene Glycol (PEG)-Based Systems

**Figure 6:**
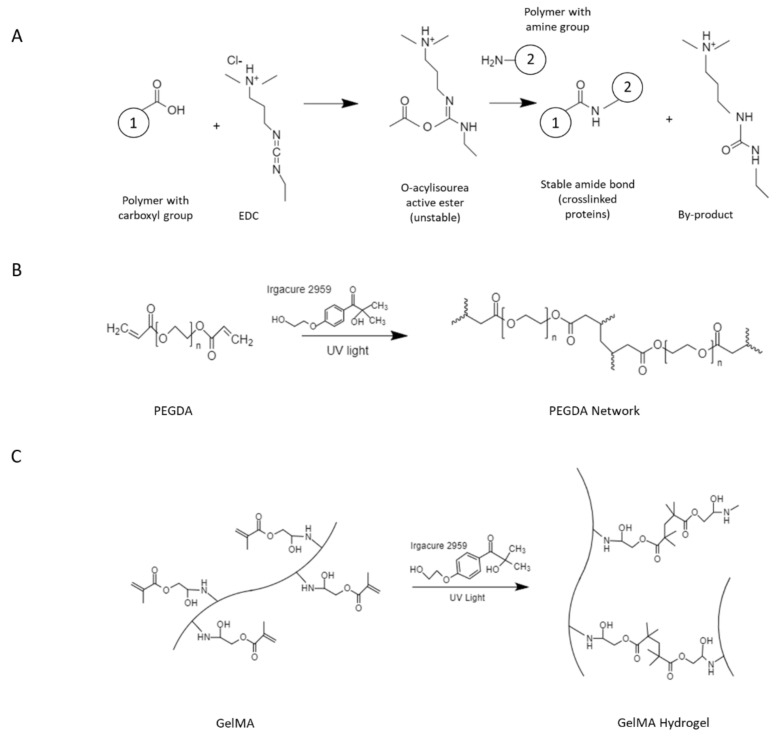
Hydrogel fabrication through chemical crosslinking via: (**A**) 1-(3-dimethylaminopropyl)-3-ethylcarbodiimide hydrochloride (EDC chemistry), and (**B**) and (**C**) UV photopolymerization using Irgacure 2959.

PEG-based hydrogels have been employed for the fabrication of biological systems due to their exceptional biocompatibility and resistance to protein adhesion [65] (Figure 5). The introduction of functional groups can yield PEG derivatives such as polyethylene glycol diacrylate (PEGDA) (Figure 6) and polyethylene glycol dimethacrylate (PEGDM), which can be chemically crosslinked to produce durable matrices [66,67] that allow tethering or embedding of biomolecules that promote proper tissue regeneration [68] (Figure 6). 

**Table 2:** Recent in vivo studies on synthetic/semi-synthetic hydrogels for diabetic wound healing.

Ref.	Year	Polymer Source and Material	Additional Functional Component(s)	Synthesis Method	Diabetic Model	Therapeutic Effect
[69]	2022	Methacrylate gelatin (GelMA)/PEGDA microneedle patch	Tazarotene (1 mg/10 mL) and exosomes (100 µg/mL) from human umbilical vein endothelial cells (HUVECs)	Photopolymerization with lithium acylphosphinate salt (LAP 0.05%, g/mL)	STZ-induced male C57BL mice	Accelerated collagen deposition, epithelial regeneration, and angiogenesis.
[70]	2022	PLGA-PEG-PLGA thermosensitive hydrogel	Copper-based MOFs containing curcumin and metformin hydrochloride	Thermal gelation	STZ-induced male BALB/c mice	Significant reduction of oxidative stress; enhanced cell migration, neovascularization, and collagen formation.
[71]	2022	Injectable hydrogel prepared from 4,5-imidazoledicarboxylic acid, zinc nitrate hexahydrate, deferoxamine mesylate and glucose oxidase (GOX)	Deferoxamine mesylate (DFO, 8.3 µg/mL)	Phase- transfer-mediated programmed GOX loading	STZ-induced female BALB/c mice	Release of zinc ions and DFO resulted in enhanced antibacterial and angiogenic effect. Significant induction of re-epithelialization and collagen deposition.
[72]	2022	PDLLA-PEG-PDLLA (25% *w*/*v*) thermosensitive hydrogel	Prussian blue nanoparticles (PBNPs, 333.3 µg/mL and 666.6 µg/mL)	Thermal gelation	STZ-induced C57BL/6J mice	Decreased reactive oxygen species (ROS) production as well as IL-6 and TNF-α levels. PBNPs dose-dependent accelerated wound closure.
[73]	2022	pH/glucose dual responsive hydrogel prepared from dihydrocaffeic acid and L-Arginine co-grafting chitosan, phenylboronic acid and benzaldehyde difunctional polyethylene glycol-co-poly(glycerol sebacic acid) and polydopamine-coated graphene oxide (GO)	Metformin (2 mg/mL)	Double dynamic bond of a Schiff-base and phenylboronate ester	STZ-induced Sprague−Dawley rats	Antibacterial properties, tissue adhesion, hemostasis. Decreased inflammatory response. Increased wound closure ratio, re-epithelialization, and regeneration of blood vessels.
[74]	2022	Supramolecular guanosine-quadruplex hydrogel	Hemin (0.36–0.54 mg) and GOX (0.125–0.5 mg)	Self-assembled gelation	STZ-induced male BABL/c mice	Significantly faster antibacterial effect, relative to commercial antibiotic. Decreased glucose concentration in the wound.
[75]	2022	Chitosan/polyvinyl acetate heterogeneous hydrogel	Human epidermal growth factor (EGF)-loaded nanoparticles, polyhexamethylene biguanide, and perfluorocarbon emulsions	Freeze-thaw cycling	STZ-induced Sprague-Dawley rats	High antibacterial and anti-inflammatory effect. Enhanced collagen production and wound closure efficiency, relative to commercial dressings.
[76]	2022	Double-layered GelMA-PLL hydrogel	Vascular endothelial growth factor (VEGF)-mimetic peptide	Photopolymerization with lithium acylphosphinate salt (LAP)	STZ-induced Sprague-Dawley rats	Enhanced antibacterial and wound-healing effect. Improved collagen deposition, angiogenesis, and re-vascularization.
[77]	2022	Oxidized alginate / platelet-rich plasma (PRP) fibrin hydrogel		Ionic crosslinking with 1.22 M CaSO_4_·2H_2_O	Male db/db (BKS.Cg-m+/+Leprdb/J) mice	Accelerated wound maturation and closure.
[78]	2022	PTFE/PU patch	Calcium-alginate hydrogel microparticles (MPs) containing *Chlorella vulgaris* and *Bacillus licheniformis*	MP encapsulation in porous PTFE membrane (inner lining) and a transparent PU film (back lining)	STZ-induced mice	Enhanced wound healing effect: 50% wound closure by day 3, and full wound closure on day 12.
[79]	2021	GelMA (10% *w*/*v*) hydrogel	Bioactive glass particles loaded with cerium (1% *w*/*v*)	Photopolymerization with LAP (0.1% *w*/*v*)	STZ-induced Sprague-Dawley rats	Wound closure of almost 95% on day 21.
[80]	2021	Cecropin-modified hyaluronic acid/ oxidized dextran / PRP composite hydrogel		Schiff base reaction	Male db/db mice	Accelerated healing of infected wounds. Shortened inflammatory stage. Increased mature collagen content.
[81]	2021	Pluronic F-127 (20%) hydrogel	Ag nanocubes with virus-like mesoporous silica containing gentamicin	Thermal gelation	STZ-induced Kunming mice	Bacterial infected wounds were fully healed by day 20, with enhanced collagen production.
[82]	2021	Carboxymethyl chitosan/poly(dextran-g-4-formylbenzoic acid) hydrogel	Peptide-modified PAN nanofibers	Schiff base reaction	Diabetic ICR mice	Enhanced antibacterial and angiogenic effect. Reduced inflammatory response. Wound closure > 96% at day 14.
[83]	2021	Hydroxyl propyl methyl cellulose (2% *w*/*w*) hydrogel	Lipid nanoparticles loaded with Valsartan (1% *w*/*w*)	Thermal gelation	STZ-induced male Sprague-Dawley rats	Enhanced healing response mediated through COX-2, NF-κB, NO, TGF-β, MMPs and VEGF pathways.
[84]	2021	Polyacrylamide/gelatin/ε-polylysine composite hydrogel		Free-radical polymerization	STZ-induced male Sprague-Dawley rats	Increased granulation tissue formation, collagen deposition, and angiogenesis. Enhanced antibacterial effect.
[85]	2021	Conductive hydrogel made from acrylamide-co-polymerized ionic liquid (VAPimBF4) and konjac glucomannan		Chemical crosslinking (EDC/NHS chemistry)	STZ-induced male Kunming mice	Highest wound healing rate when coupled with electrical stimulation. Increased antibacterial effect, Col-1 production, and new vessel growth.
[86]	2021	N-carboxyethyl chitosan/adipic acid dihydrazide pH responsive hydrogel	Insulin (0.67 U/mL)	Crosslinking by hyaluronic acid-aldehyde (imine and acylhydrazone bonds)	STZ-induced male Sprague-Dawley rats	Significant reduction of glucose levels in the wound. Decreased inflammation phase. Increased granulation tissue formation, collagen deposition, and re-epithelialization.
[87]	2021	Quaternized chitosan/oxidized hyaluronic acid self-healing hydrogel	α-lipoic acid-loaded MOFs	Schiff base reaction	STZ-induced male Sprague-Dawley rats	Increased collagen deposition, cell proliferation and neovascularization. Accelerated wound healing.
[88]	2021	Chitosan/polyvinyl acetate hydrogel	Chitosan nanoparticles loaded with human epidermal growth factor (EGF, 60 µg/mL) and Ag^+^ ions	Freeze-thaw cycling	STZ-induced Sprague-Dawley rats	Remarkable antibacterial effect. Enhanced tissue maturation and wound closure: 40% on day 3, and 97% on day 14.
[89]	2021	Pluronic F-127 (20% *w*/*v*) hydrogel	Sodium ascorbyl phosphate (400 μM) and Wharton’s jelly mesenchymal stem cells (WJMSC)	Thermal gelation	STZ-induced male Sprague-Dawley rats	Shortened inflammatory response. Improved dermis regeneration, neovascularization, and collagen deposition.
[90]	2020	Supramolecular hydrogel based on ferrocene, hyaluronic acid, β-cyclodextrin, and rhein		Intermolecular π−π interactions and hydrogen bonds	STZ-induced C57 mice	Anti-inflammatory properties of rhein facilitated transition from the inflammatory phase into the proliferation phase, thus, favoring normal wound healing.
[91]	2020	Pluronic F-127 hydrogel	Exosomes derived from human umbilical cord MSCs (300 μg/mL)	Thermal gelation	STZ-induced male Sprague-Dawley rats	Increased vascularization of wound granulation tissue, shortening wound healing time. Improved epithelial regeneration.
[92]	2020	4-carboxybenzaldehyde-PEG/glycol chitosan/silk fibroin/PRP self-healing hydrogel		Schiff base reaction + crosslinking with 10% calcium gluconate	STZ-induced Sprague-Dawley rats	Enhanced angiogenesis, re-epithelialization, nerve repair, and wound healing rate.
[93]	2020	Chitosan/polyurethane hydrogel membrane	Bone marrow mononuclear cells (1 × 10^6^) injected into the edge of the wound prior to hydrogel application	Chemical crosslinking (urea/urethane bonds)	STZ-induced female Wistar rats	Hemostatic and anti-inflammatory effect. Wound closure > 90% after 14 days.
[94]	2020	Stimuli-responsive supramolecular hydrogel made from polyvinyl alcohol/N-carboxyethyl chitosan/agarose/Ag nanowires		Hydrogen bonding	STZ-induced male Sprague-Dawley rats	Bactericidal effect. Promoted angiogenesis and collagen deposition. Accelerated wound healing rate.
[95]	2020	Poly(N-isopropyl-acrylamide)/poly(γ-glutamic acid) hydrogel (20 mg/mL total concentration)	Superoxide dismutase (2 mg/mL)	Thermal gelation	STZ-induced male Sprague-Dawley rats	Reduced inflammation. Enhanced collagen production and epidermal formation.
[96]	2020	N-carboxyethyl chitosan (7.5% *w*/*v*)/adipic acid dihydrazide (7.5% *w*/*v*)/hyaluronic acid-aldehyde (5% *w*/*v*) composite hydrogel	Encapsulated bone marrow mesenchymal stem cells (2 × 10^5^)	Crosslinking by hyaluronic acid-aldehyde (imine and acylhydrazone bonds)	STZ-induced male Sprague-Dawley rats	Inhibited chronic inflammation. Enhanced formation of granulation tissue, cell proliferation and neovascularization.
[97]	2020	γ-polyglutamic acid (0.5 g/mL) hydrogel	Human cell-free fat extract (5 mg/mL)	Chemical crosslinking (EDC/NHS chemistry)	Male BKS-Leprem2Cd479/Nju mice	Improved cell proliferation, collagen deposition and continuous epidermal formation. Significant angiogenesis.
[98]	2020	Silk fibroin-polyvinyl pyrrolidone hydrogel	L-carnosine and curcumin	Mixing/vortex shearing (physical crosslinking)	STZ-induced BALB/c mice	Significant antibacterial and anti-inflammatory effect. Enhanced wound healing.
[99]	2020	[2-(methacryloloxy)ethyl]dimethyl-(3-sulfopropyl) ammonium hydroxide (SBMA)/2-Hydroxyethyl methacrylate (HEMA) and 3-[[2-(Methacryloyloxy)ethyl] dimethylammonio] propionate (CBMA)/HEMA zwitterionic cryogels	miRNA146a-conjugated cerium oxide nanoparticles	Free-radical polymerization with 13.6 mg/mL ammonium persulfate	Db/Db female mice	Full wound healing on day 14. Downregulation of inflammatory markers. Increased Col1a2 expression.
[100]	2020	Polyvinyl alcohol (8% *w*/*v*)/sodium alginate (1% *w*/*v*) hydrogel	Green tea polyphenol nanoparticles	Ionic crosslinking (CaCl_2_, 100 μg/mL) and hydrogen bonding	STZ-induced female Sprague-Dawley rats	Increased granulation tissue formation and collage deposition. Accelerated wound healing.
[101]	2019	Chitosan/PEG hydrogel	Ag nanoparticles	Chemical crosslinking with glutaraldehyde	Alloxan-induced rabbits	Increased bactericidal effect. Accelerated re-epithelialization and collagen deposition. Full wound closure on day 14.
[102]	2018	A5G81-modified poly(polyethylene glycol cocitric acid-co-N-isopropylacrylamide) hydrogel		Thermal gelation	B6.BKS(D)-Lepr^db^/J mice	Enhanced re-epithelialization and granulation tissue formation. Faster wound closure than that achieved with commercial dressings.
[103]	2018	Hyperbranched PEG/thiolated hyaluronic acid injectable hydrogel	Encapsulated adipose-derived stem cells (2.5 × 10^6^ cell/mL)	thiol-ene click reaction	STZ-induced male Sprague-Dawley rats	Reduced inflammatory response. Increased angiogenesis and re-epithelialization.
[104]	2017	Polymethyl methacrylate/Polyvinyl alcohol hydrogel particles	Collagen, Ag nanowires, and chitosan	UV photocrosslinking (Irgacure 184)	STZ-induced male Wistar ratsSTZ-induced Landrace pigs	Enhanced collagen production and epidermal cell migration. Reduced inflammatory response.
[105]	2017	Phenylboronic-modified chitosan (1.2 wt%)/poly(vinyl alcohol) (0.6 wt%)/benzaldehyde-capped PEG (0.6 wt%) hydrogel	Insulin (0.3 wt%) and L929 fibroblasts (1.2 × 10^6^ cells/mL)	Schiff base reaction	STZ-induced Sprague-Dawley rats	Improved control of glucose levels in wound. Increased neovascularization and collagen deposition. Enhanced wound closure rate.
[106]	2016	Sodium carboxymethylcellulose/propylene glycol hydrogel	*Blechnum orientale* extract (2–4% wt)	Hydrogen bonding	STZ-induced male Sprague-Dawley rats	Significant bactericidal and antioxidative effect. Enhanced re-epithelialization, fibroblast proliferation, collagen synthesis, and angiogenesis.
[107]	2016	Gelatin methacrylate (15% *w*/*v*) hydrogel	Desferrioxamine (1% *w*/*v*)	UV photocrosslinking with Irgacure 2959 (0.5% *w*/*v*)	STZ-induced male Sprague-Dawley rats	Accelerated neovascularization, granulation tissue remodeling, and wound closure.

In the context of wound healing, Xu et al., fabricated an in situ polymerizable hydrogel encapsulating adipose-derived stem cells to promote skin regeneration in murine diabetic wounds. By means of a reversible addition−fragmentation chain-transfer (RAFT) polymerization mechanism, hyperbranched multi-acrylated PEG macromers were synthesized and further combined with thiolated hyaluronic acid to produce a hydrogel via thiol-ene click reaction [103]. The results from in vivo testing revealed the therapeutic effect of the proposed system, as evidenced by accelerated healing rates and granular tissue formation, in addition to reduced inflammation. 

Several factors contribute to the chronicity associated with DFUs, being oxidative stress one of the most detrimental elements for diabetic wound healing. For this reason, a lot of research efforts are currently focusing on the development of materials with scavenging properties that can protect the wound bed from the mitochondrial damage inflicted by reactive oxygen species (ROS). In this sense, the recent work conducted by Xu et al. involved the fabrication of a poly(d,L-lactide)-PEG-poly(d,L-lactide) (PLGA-PEG-PLGA) hydrogel for the encapsulation and controlled release of Prusian blue nanoparticles (PBNPs), a synthetic material that can mimic the ROS scavenging activity of naturally occurring antioxidant enzymes, such as peroxidase, catalase, and superoxide dismutase [72]. The thermosensitive properties of the prepared hydrogel allowed in situ gelation at physiological temperature. Furthermore, in vivo experiments on murine diabetic wound models demonstrated the ROS scavenging features of PBNPs, as confirmed by dihydroethidium staining. This in turn favored an anti-inflammatory response that mitigated chronic inflammation and thus, enhanced proper wound healing and neovascular remodeling, relative to the control treatment without PBNPs. 

Moreover, the studies conducted by Yang and coworkers aimed at the design of a multifunctional hydrogel system that simultaneously addressed the regulation of blood glucose and oxidative stress levels in diabetic ulcers [70]. To this end, the authors developed copper-based metal-organic frameworks (MOFs) by a solvothermal method, which could be loaded with metformin hydrochloride (MH), a hypoglycemic and hydrophilic drug, as well as curcumin, a natural lipophilic agent that has been previously shown to have ROS scavenging properties [108]. The resulting MOFs exhibited ~65% drug entrapment efficiency and were further embedded into PLGA-PEG-PLGA thermosensitive hydrogels for the controlled and sustained release of the therapeutic agents, using a type 1 diabetes wound model. It was observed that blood glucose levels showed a continuously declining trend from day 5 until the last day of treatment (day 20), whereas the antioxidative capacity of the hydrogel was validated through a sensitive chemiluminescent of L-012. Wound analysis revealed that the proposed composite dressing promoted cell proliferation, neovascularization, and collagen formation, reaching >90% wound closure after 20 days.

Finally, Pluronics or Poloxamers are a family of polyethylene oxide/polypropylene oxide/polyethylene oxide (PEO-PPO-PEO) triblock copolymers which are non-toxic and FDA-approved for biomedical use (Figure 5). The PPO portion acts as the hydrophobic component, whereas the PEO segments provide hydrophilicity to the material. Within this family of polymers, Pluronic F-127 hydrogels have increasingly drawn attention, as they can be safely used as synthetic platforms for the delivery of drugs and cells through in situ gelation at body temperature [109,110,111]. For instance, Jiao et al. recently evaluated the effect of Wharton’s jelly mesenchymal stem cells (WJMSCs) encapsulated in a Pluronic F-127 hydrogel (20% *w*/*v*) on the healing process of a type 2 diabetes wound model [89]. Prior to encapsulation, the hydrogel precursor was supplemented with sodium ascorbyl phosphate to increase WJMSC viability. The results showed that the proposed hydrogel dressing significantly stimulated collagen deposition, re-epithelialization, and anti-inflammatory response 14 days post-transplantation. Moreover, WJMSC engraftment in the wound was also evidenced, which confirmed the immunomodulatory and regenerative potential of this hydrogel system for DFU treatment.

#### 4.2.2. Polyvinyl Alcohol (PVA)-Based Systems

PVA is a hydrophilic polymer with biocompatible, biodegradable, and semi-crystalline features that have been of great interest in the biomedical field [112] (Figure 5). PVA can be physically crosslinked by repeated freeze-thaw cycles (cryogelation), or chemically crosslinked using glutaraldehyde or epichlorohydrin. Both synthesis routes render PVA hydrogels with remarkable hydrophilicity and chemical stability. In cryogelation, as the temperature of the PVA solution decreases, more ice crystals are formed, which in turn causes the PVA polymer chains to be “pushed out” as impurities, increasing their concentration in the unfrozen liquid phase. This phenomenon enables physical interactions among PVA molecules and the eventual creation of PVA crystallites. Such crystallites are not soluble in water and, therefore, remain after thawing, so that at the end of repeated freeze-thaw cycles, this yields a porous hydrogel [113,114]. In this regard, Takei et al. used a cryogelation technique to incorporate PVA into a chitosan-gluconic acid conjugate (GC) hydrogel, towards improving the mechanical properties, water retention capacity, and resistance to enzyme degradation of pure GC hydrogels. The produced dressings were also loaded with basic fibroblast growth factor (bFGF) and then evaluated on partial-thickness diabetic wounds. The authors reported that hydrogel implantation contributed to a high expression of inflammatory cells and the secretion of chemical mediators that accelerated wound healing, avoiding the formation of fibrotic tissue [115].

Furthermore, PVA-based hydrogels have been recently tested for the stabilization and sustained release of natural biomolecules with antioxidant potential, such as green tea-derived polyphenols (TPs). In their studies, Chen et al., explored the fabrication of a PVA-alginate composite system, whose synthesis involved a combination of ionic crosslinking and hydrogen bonding to encapsulate TPs nanoparticles [100]. The therapeutic effect of the proposed dressing was evaluated on excisional linear wounds in diabetic rats for seven days. As reported by the authors, the designed hydrogel system promoted higher collagen deposition and maturation as well as granulation tissue formation, relative to the controls (hydrogel without TPs and hydrogel with powdered TPs). Finally, an analysis of the PI3K/AKT protein pathway, which has been associated with cell proliferation, angiogenesis and glucose metabolism [116,117] allowed to confirm that TPs helped regulate such pathway, which translated into a significant improvement of the wound healing process [118].

Another remarkable example of the development of multifunctional semi-synthetic hydrogels for DFU applications is the study conducted by Zhao et al. The authors set out to create a dressing that enabled tissue restoration, while allowing controlled in situ degradation, avoiding the neo tissue damage and patient discomfort that is commonly observed upon dressing replacement [94]. In brief, stimuli-responsive 3D supramolecular structures were fabricated through hydrogen bonding using PVA, agarose, N-carboxyethyl chitosan and silver nanowires. The incorporation of Na_2_B_4_O_7_ into the hydrogel precursor solution granted the final material the ability to degrade upon exposure to heat, pH reduction, ultrasound, or diol-containing molecules, such as glucose. This biodegradability was attributed to the presence of reversible boronate-based reactions between PVA and Na_2_B_4_O_7_, which can be affected by the factors mentioned above. Furthermore, in vivo testing of this scaffold revealed strong bactericidal traits, and after 20 days of treatment, foot wounds in diabetic rats were almost fully healed, versus approximately 35% of wound closure observed in the control group (untreated). 

#### 4.2.3. Gelatin-Based Systems

Gelatin has been studied as a delivery vehicle for the transport of bioactive agents, such as cytokines and growth factors secreted by mesenchymal stem cells, which contribute to tissue repair and play an important role in wound healing by regulating the wound environment and stimulating cell migration, adhesion, and proliferation. This results in enhanced chemotactic responses, which are very much needed in a diseased cell environment such as DFUs. For instance, Yoon et al. developed an enzyme-catalyzed gelatin hydrogel as a sprayable dressing material capable of delivering cell-attracting chemokines for diabetic wound healing [63]. The gelatin-hydroxyphenyl propionic acid hydrogels were prepared in situ by crosslinking the phenol moieties with horseradish peroxidase and H_2_O_2_, while simultaneously loading the material with chemotactic cytokines (IL-8, 5 µg/mL or MIP-3α, 10 µg/mL). Diabetic mice were treated with the fabricated dressing, and the observed results showed accelerated cell infiltration into the wound area, which in turn promoted wound healing, neovascularization, and increased collagen deposition.

Additionally, the studies reported by Chen et al. described the development of a gelatin methacrylate (GelMA) system (Figure 5 and Figure 6) using in situ photo-crosslinking to obtain hydrogels coupled with desferrioxamine (DFO), an angiogenic drug that supports wound healing. As evidenced by the results, the proposed hydrogel dressings enhanced blood vessel network formation, which in turn ensured sufficient oxygen and nutrient delivery into the wound area. Ultimately, these GelMA scaffolds promoted granulation and epithelial tissue generation, as well as a favorable cell microenvironment that led to effective diabetic wound treatment [107]. More recently, Yuan and coworkers designed a novel microneedle patch system based on a GelMA/PEGDA composite matrix [69]. The rationale behind their studies was that the incorporation of gel microneedles on the inner side of the wound dressing could significantly improve transdermal delivery of target biological agents, without the pain associated with local injection approaches. Towards this end, tazarotene, a medication known for its ability to stimulate collagen production and angiogenesis, was mixed with the GelMA/PEGDA precursor solution by means of grafted isocyanatoethyl acrylate-modified β-cyclodextrin (β-CD-AOI_2_) to improve the water solubility of the drug. After this, exosomes (100 µg/mL) derived from human umbilical vein endothelial cells (HUVECs) were added to the mixture and further poured into a vacuum mold for microneedle formation. Finally, the hydrogel dressing was obtained through photopolymerization with lithium acylphosphinate salt (0.05%, g/mL). Following in vitro assays that confirmed the sustained and controlled delivery of exosomes and tazarotene, in vivo testing was performed on a murine diabetic wound model. The results showed that the hydrogel loaded with both, tazarotene and HUVEC-exos, elicited a significantly enhanced wound healing response in terms of cell migration, angiogenesis, and wound closure rate, relative to the blank hydrogel (pure GelMA/PEGDA), or the hydrogel loaded with tazarotene only. The presence of PEGDA in the matrix ensured increased crosslink density, and thus, desired mechanical stability for the developed system.

#### 4.2.4. Chitosan-Based Systems

Because of its remarkable antibacterial, anti-inflammatory, and hemostatic features [119,120,121], chitosan has been continuously employed as a base material for the synthesis of semi-synthetic hydrogel dressings with advanced biological and physical properties. For instance, the experiments conducted by Mirhamed et al., demonstrated the potential of chitosan-derived hydrogels for preserving the bioactivity of key growth factors, ensuring their sustained release into a wound. Through carbodiimide conjugation reaction, recombinant human epidermal growth factor (rhEGF) was conjugated to sodium carboxymethyl chitosan (NaCMCh), with the aim of protecting rhEGF from the proteolytic degradation that occurs in chronic wound environments. These conjugates were further embedded into a NaCMCh/polyvinylpyrrolidone (PVP) hydrogel. The authors were able to show that the developed dressing maintained rhEGF functionality over time, and its controlled release and degradation significantly increased rhEGF therapeutic effect. Moreover, in vivo evaluation of this hydrogel in diabetic rats proved that it indeed stimulated fast wound closure, re-epithelialization, as well as granulation tissue formation [122].

Additional studies on chitosan-derived 3D wound dressings have sought to improve the mechanical behavior of the synthesized material as well. In this sense, Kamel et al. prepared hydrogel-based scaffolds from the combination of hydroxypropyl methylcellulose (HPMC) with high-viscosity chitosan. This mixture was then enriched with pioglitazone HCl (PG), a known anti-diabetic drug. The hydrogel was freeze-dried and further used to treat diabetic wounds in rats. The authors highlighted the improved flexibility and adhesiveness of the scaffold, which could be attributed to the presence of HPMC. Moreover, relative to the control (untreated) group, the positive effect of the HPMC-chitosan dressing was evidenced by a faster wound healing rate, as well as a reduction in matrix metalloproteinase-9 (MMP9) and tumor necrosis factor (TNF-α) levels in the wound [123].

More recently, Lee and coworkers proposed the fabrication of a novel multifunctional DFU hydrogel system to simultaneously address several issues of the diabetic wound environment, and thus, provide a more effective treatment alternative [75]. By means of a freeze-thaw cycling method (−20 to 25 °C), the authors prepared heterogeneous chitosan/ polyvinyl acetate dressings containing the following: (1) a perfluorocarbon emulsion to favor oxygen transport into the wound; (2) EGF-loaded nanoparticles to stimulate cell adhesion and proliferation; and (3) polyhexamethylene biguanide (PHMB), an antimicrobial and antiviral agent to prevent infection. Material characterization of the synthesized hydrogel revealed that the nanoparticles and the emulsion also served as mechanical reinforcement elements for the scaffold, whereas in vitro evaluation demonstrated a remarkable bactericidal effect against *S. aureus* and *S. epidermidis*. Upon performing in vivo experiments in diabetic rats, the developed composite dressing offered an improved wound closure rate and reduced inflammatory response, relative to treatment with gauze or a commercially available dressing (HeraDerm).

#### 4.2.5. Plasma-Based Systems

Blood plasma has been reported to be a promising material source for the design of bioactive matrices, due primarily to its biochemical composition and its autologous nature [124]. Blood plasma contains platelets, glycoproteins, immunoglobulins, and growth factors that swiftly adhere and aggregate at the injury site, preventing excessive bleeding and subsequent vascular complications [125]. Specifically, platelets play a key role in the hemostatic system since they are degranulated of their secretory granules, which grants them the capacity to stimulate tissue repair, blood vessel regeneration, and cell differentiation [126].

Platelet-rich plasma (PRP) is a type of autologous plasma with high platelet concentration [127], which is considered a natural gelling sealant that works as a drug vehicle for the release of essential platelet-derived growth factors, such as: platelet-derived growth factor (PDGF) (AA, BB, and AB isomers), transforming growth factor-β (TGF-β), platelet factor 4 (PF4), interleukin-1 (IL-1), platelet-derived angiogenesis factor (PDAF), vascular endothelial growth factor (VEGF), epidermal growth factor (EGF), epithelial cell growth factor (ECGF), and insulin-like growth factor (IGF), among others [128,129]. These factors support the wound healing process by (i) stimulating cells around the wound area to participate in the restoration of the affected tissue; and (ii) promoting cell differentiation through the control of cytokine release and macrophage activity, reducing inflammation and accelerating tissue regeneration in chronic environments such as DFUs.

Current studies have reported the successful incorporation of PRP into the development of hydrogel dressings for DFU treatment. For instance, Qian et al., designed a composite system from silk fibroin, glycol chitosan (GCTS), and PRP, which was eventually tested in a type 2 diabetes rat model. In short, PEG was modified with 4-carboxybenzaldehyde (CB) through the esterification reaction of carboxyl with hydroxyl to produce CBPEG, which could crosslink GCTS through a reversible Schiff base reaction [92]. Moreover, silk fibroin was added to provide mechanical reinforcement to the scaffold. The authors evaluated the in vitro and in vivo performance of this composite hydrogel, finding that it allowed for a more controlled degradation of the PRP present in the matrix, as well as a more sustained delivery of the bioactive factors associated with PRP when compared to a pure PRP hydrogel. This was attributed to the resistance to enzymatic degradation of chitosan and silk fibroin. Altogether, the biological properties of this novel hydrogel resulted in accelerated wound healing, characterized by increased collagen deposition, granulation tissue formation, re-epithelialization, and nerve regeneration.

More recently, the experiments conducted by Wei et al., focused on the creation of a hydrogel with enhanced bactericidal features towards regulating chronic inflammation in diabetic wound environments [80]. In this sense, dextran and hyaluronic acid (HA) were chosen as the base materials, which underwent chemical modification prior to hydrogel synthesis. Essentially, cecropin, an antimicrobial peptide (AMP), was tethered to HA through EDC/NHS chemistry (amide bond formation) to yield an HA-AMP polymer, while dextran was oxidized (ODEX) with sodium periodate (NaIO_4_) and diethylene glycol to add aldehyde groups. Final hydrogel fabrication was achieved through a Schiff’s base reaction between the aldehyde groups in ODEX and the amino groups in HA-AMP and PRP. A control hydrogel was also prepared, which contained everything but PRP. The authors observed that both types of hydrogels exhibited significant antibacterial properties against *P. aeruginosa* and *S. aureus* in infected diabetic wounds (mouse) and also promoted downregulation of inflammatory factors, such as TGF- β1, TNF- α, IL-6 and IL-1β. Nonetheless, the hydrogel containing PRP exhibited greater VEGF expression, confirming the angiogenic potential of PRP. Furthermore, following a similar oxidation procedure to modify alginate, Garcia-Orue and coworkers set out to develop a biodegradable matrix that could deliver the relevant growth factors present in PRP to promote wound healing without the need to change the dressing after use [77]. Oxidation degree, and thus, the biodegradability of the final hydrogel could be tightly controlled by adjusting the amount of NaIO_4_ utilized for alginate oxidation. Oxidized alginate (2.5%)-PRP hydrogels were produced by ionic crosslinking using CaSO_4_, along with a control hydrogel lacking PRP. They were then applied to wounds in diabetic mice for 15 days. Interestingly, even though wound healing appeared enhanced in both sample groups (hydrogels with or without PRP), no significant differences were found between them. This result disagreed with what had been previously observed during in vitro testing with human fibroblasts and keratinocytes, in which the PRP-containing hydrogels displayed increased cell adhesion and proliferation, relative to the control hydrogel. The authors attributed this inconsistency between in vitro and in vivo tests to the fact that the PRP used was of human origin, and therefore, had a positive effect when tested with human cells, but such effect could have been hindered by its xenogeneic nature when applied to mice. 

## 5. Conclusions

Diabetic foot ulcers are a serious health condition that poses a significant risk to the patient and a great financial burden to the healthcare system. Impaired healing in DFUs is a complex and multifactorial problem since diabetic wounds are characterized by high levels of blood glucose and reactive oxygen species, infection, abnormal inflammatory response, as well as hindered angiogenesis. Currently, the development of a successful clinical treatment for DFUs remains a challenge for the scientific community. Nonetheless, extensive research efforts are continuously made towards elucidating the key combination of elements that could allow for the fabrication of a truly effective DFU treatment. 

Due to their inherent properties, hydrogels are attractive materials for the design of optimal DFU wound dressing systems. Hydrogels are 3D platforms that not only can provide skin-like mechanical properties and a moist environment for the wound bed, but also can serve as vehicles for the delivery of biological cues that are essential for adequate wound repair. In this context, natural and synthetic polymers have been explored for the preparation of pure or composite hydrogel dressings that can additionally transport a myriad of relevant bioactive agents, such as antibiotics, antioxidants, stem cells, PRP, growth factors, and insulin, among others. Even though natural polymers such as chitosan, alginate, and gelatin can intrinsically help ameliorate the defective conditions of the diabetic wound environment, there currently appears to be an agreement on the need to design semi-synthetic hydrogels that can strategically take advantage of the traits offered by natural polymers (biodegradability, biocompatibility, bioactivity, and antibacterial properties), while incorporating the unique versatility and reproducibility provided by synthetic polymers. For instance, nano-hydrogels embedded with quercetin and oleic acid have recently shown promising results in a clinical trial, by reducing wound healing time without adverse effects in 28 DFU patients [130]. 

Furthermore, because DFUs are a multifactorial problem, the growing body of scientific data in the field seems to indicate that the answer to this problem could be the development of multifunctional hydrogel dressings; in other words, 3D polymeric systems that can simultaneously address several issues of the diabetic wound, ideally in the absence of additional chemicals (crosslinkers) that could compromise the cytocompatibility of the scaffold. Moreover, based on the cumulative results from multiple in vivo studies recently reported, the delivery of antioxidants and antibacterial agents, as well as bioactivity to stimulate desired cell infiltration and proliferation, appear to be determinant factors in the success of clinically viable DFU hydrogel treatments.
